# Regulation and Migratory Role of P-Selectin Ligands during Intestinal Inflammation

**DOI:** 10.1371/journal.pone.0062055

**Published:** 2013-04-22

**Authors:** Ute Hoffmann, Matthias Pink, Uta Lauer, Markus M. Heimesaat, Caroline Winsauer, Andrei Kruglov, Kerstin Schlawe, Claudia Leichsenring, Oliver Liesenfeld, Alf Hamann, Uta Syrbe

**Affiliations:** 1 Deutsches Rheumaforschungszentrum, Berlin, Germany; 2 Institut für Mikrobiologie und Hygiene, Campus Benjamin Franklin, Universitätsmedizin Charité, Berlin, Germany; 3 Klinik für Gastroenterologie, Infektiologie und Rheumatologie, Campus Benjamin Franklin, Universitätsmedizin Charité, Berlin, Germany; Johannes Gutenberg University of Mainz, Germany

## Abstract

Dendritic cells from mesenteric lymph nodes (MLN) can convert retinal to retinoic acid (RA), which promotes induction of the gut-specific homing receptor α4β7. In contrast, priming within peripheral lymph nodes leads to upregulation of E- and P-selectin ligands (E- and P-lig). Apart from its α4β7 promoting effect, RA was shown to suppress E- and P-lig induction in vitro. However, enhanced frequencies of P-lig^+^ CD4^+^ T cells were reported during intestinal inflammation. To understand this contradiction, we first determined whether location of intestinal inflammation, that is, ileitis or colitis, affects P-lig induction. Both conditions promoted P-lig expression on CD4^+^ T cells; however, P-lig expressed on T cells facilitated Th1 cell recruitment only into the inflamed colon but not into inflamed small intestine induced by oral *Toxoplasma gondii* infection. A majority of P-lig^+^CD4^+^ T cells found within MLN during intestinal inflammation co-expressed α4β7 confirming their activation in the presence of RA. Mesenteric P-lig^+^CD4^+^ cells co-expressed the 130 kDa isoform of CD43 which requires activity of core 2 (beta)1,6-N-acetyl-glycosaminyltransferase-I (C2GlcNAcT-I) suggesting that C2GlcNAcT-I contributes to P-lig expression under these conditions. To test whether inflammatory mediators can indeed overrule the inhibitory effect of RA on P-lig expression we stimulated CD4^+^ T cells either polyclonal in the presence of IL-12 and IFNγ or by LPS-activated MLN-derived dendritic cells. Both conditions promoted P-lig induction even in the presence of RA. While RA impeded the induction of fucosyltransferase-VII it did not affect IL-12-dependent C2GlcNAcT-I induction suggesting that C2GlcNAcT-I can support P-lig expression even if fucosyltransferase-VII mRNA upregulation is dampened.

## Introduction

Effector/memory T lymphocytes recirculate through peripheral tissues and by that provide immune surveillance of the body. Homing receptors required for their recruitment into peripheral tissue are induced upon effector cell differentiation. *In vivo* experiments using ovalbumin-TCR-transgenic CD4^+^ T cells and systemic administration of ovalbumin with LPS as adjuvant showed that the priming location of the naive T cells dictates the induced homing molecule pattern: T cells activated within peripheral lymph nodes (PLN) upregulate E- and P-selectin ligands (E- and P-lig), whereas T cells activated within mesenteric lymph nodes (MLN) express α4β7 and CCR9 [1]. E- and P-selectin are displayed by skin vessels and function as skin-specific gate keepers whereas mucosal addressin cell adhesion molecule (MAdCAM) and the chemokine CCL25 are displayed by vessels within the intestine where they control T cell entry into the gut [2].

Tissue-specific antigen-presenting cells (APC), in particular CD103^+^ dendritic cells (DCs), but also stromal cells are instrumental for the tissue-specific induction of homing receptors [3]–[5]. In contrast to PLN-derived DCs, MLN-derived DCs have the capacity to convert retinal to retinoic acid (RA) which promotes induction of the gut-homing receptor α4β7 and, in cooperation with IL-4, also induction of CCR9 [6], [7].

E- and P-lig are induced by IL-12 *in vitro* resulting in high expression of selectin ligands in Th1 cells [8], [9]. *In vivo*, additional pathways seem to exist as P-lig is also expressed on Th2 cells as well as on subsets of Tregs [1], [10], [11]. Vitamin D which controls CCR10 expression in the skin has no inducing effect on P-lig expression [12].

E- and P-lig are composed of a carrier protein which requires appropriate glycosylation in order to bind to E- and P-selectin. Generation of these epitopes mainly depends on induction of core 2 (beta)1,6-N-acetyl-glycosaminyltransferase I (C2GlcNAcT-I) and, specifically in T cells, on fucosyltransferase-VII (FucT-VII) [13]–[15]. RA, apart from its promoting effect on the gut-homing molecule expression, counteracts E- and P-lig induction and was shown to suppress FucT-VII induction in T cells [4], [6], [12].

Functional binding epitopes serving as E- and P-lig are generated by specific glycosylation of several carrier proteins upon activation and differentiation of T cells. P-selectin glycoprotein ligand-1 (PSGL-1), a dimeric glycoprotein expressed constitutively on all T cells, is the predominant carrier of functional P-lig [16]. In contrast, E-lig are displayed on both PSGL-1 and alternative carrier proteins such as CD43 and CD44 [17]–[19].

Generation of P-selectin binding epitopes depends on the induction of fucosyltransferase (FucT)-VII and core 2 (beta)1,6-N-acetyl-glycosaminyltransferase I (C2GlcNAcT-I) [13]–[15]. Thus, Chinese hamster ovary (CHO) cells expressing PSGL-1 bind to P-selectin only if co-transfected with fucosyltransferases and C2GlcNAcT-I [20]. Fucosylation is required for synthesis of both, E- and P-lig. In T cells, E- and P-lig expression requires fucosyltransferase (FucT)-VII activity while FucT-IV is dispensable [15]. C2GlcNAcT-I is particularly important for P-lig generation, since leukocytes of C2GlcNAcT-I^-/-^ mice display impaired P-selectin binding while binding to E-selectin is less affected [13], [14], [21]. RA, apart from its promoting effect on the gut-homing molecule expression, counteracts E- and P-lig induction and was shown to suppress FucT-VII induction in T cells [4], [6], [12].

In line with the opposing effect of RA on the induction of α4β7 and E- and P-lig, an almost mutual exclusive expression of E- and P-lig and α4β7 is indeed observed under homeostatic conditions on CD4^+^ T cells [1], [10]. However, during experimental colitis enhanced frequencies of P-lig expressing CD4^+^ T cells were reported within intestinal sites, such as the MLN and the lamina propria [22]. Also during immunization with OVA and CFA injected intraperitoneally an IL-12-dependent upregulation of P-lig was observed on antigen-reactive T cells in the draining lymph nodes suggesting that inflammatory stimuli can overrule the tissue-specific induction pattern of selectin ligands [23].

To analyze whether P-lig induction is a common feature of intestinal inflammation we analyzed P-lig expression on CD4^+^ T cells within MLN during inflammation either of the small intestine induced by oral infection with the protozoan parasite *Toxoplasma gondii* (*T. gondii*) which induces a strong Th1 cytokine-driven pathology within the small intestine [24], [25] or during colitis. We show that under both conditions increased frequencies of P-lig^+^ T cells are present and a large proportion of these cells co-expressed α4β7.

Also *in vitro* we found that activation of MLN-derived DCs by Toll-like receptor (TLR) ligands such as LPS increased their capacity to induce P-lig, while induction of α4β7 was partially impaired. Upon polyclonal activation of CD4^+^ T cells in the absence of APCs, IL-12 and IFNγ increased P-lig induction even in the presence of RA suggesting that inflammatory mediators, like LPS, rather act via induction of IL-12 than by modulating the retinol-converting capacity of the DCs. RA impeded the activation–induced FucT-VII expression, however, it did not impair the IL-12-dependent C2GlcNAcT-I induction which apparently supports P-lig generation even in the presence of low FucT-VII mRNA expression.

## Materials and Methods

### Mice

6–12 weeks old, female BALB/c, C57BL/6, RAG-1^−/−^ and DO11.10 purchased from the Bundesinstitut fuer Risikobewertung (BfR, Berlin, Germany) were housed under specific pathogen free (SPF) conditions. C.B-17 severe combined immunodeficient (SCID) mice were obtained from Charles River Breeding Laboratories (Sulzfeld, Germany) and used at an age of 4–6 weeks. *Fut7^−/−^* mice on C57BL6 background provided by J. Lowe were backcrossed to BALB/c at least seven times. All animal experiments were performed in accordance with German animal protection laws after approval by the LaGeSo (G0227-98, G0331-08).

### Antibodies, Staining and Sorting Reagents

The following antibodies were produced in our laboratory: anti-FcR II/III (2.4G2), anti-CD4-FITC (GK1.5), anti-CD8 (53–672), anti-CD25 (PC/6), anti-Mac-1 (M1/70), anti-CD3 (145.2C11), anti-CD28 (37.51), and anti-IL4 (11B11), anti-IFN-γ (AN18.17.24), anti-IL12 (C17.8), anti α4β7-Bio (DATK32). The following antibodies were purchased from BD Pharmingen (Heidelberg, Germany): anti-CD4-FITC or anti-CD4-PerCP (RM4-5), anti-CD62L-PE (Mel-14), anti-CD45RB-PE (16A), IgG1-FITC (R3-34) and SA-PerCP. The anti-CD43-PECy7 (1B11) and isotype control were purchased from BioLegend (London, UK). The recombinant P-selectin human IgG fusion protein was kindly provided by D. Vestweber (Munster, Germany). PE-labeled anti-human IgG antibodies were obtained from Jackson Immuno Research (Suffolk, UK). All microbeads were obtained from Miltenyi Biotec (Bergisch-Gladbach, Germany).

### Induction of Intestinal Inflammation

To induce inflammation of the small intestine female C57BL/6 mice were infected per os with 10 or 100 cysts of *Toxoplasma gondii* (ME49 strain) which were obtained from NMRI mice infected 2–3 months prior. Migration studies were performed on day 3, 5 and 7 after oral infection with 100 cysts. For T cell analysis from MLN animals were orally infected with 10 cysts of Toxoplasma and sacrificed on day 9.

For induction of large intestinal inflammation (experimental colitis) either 3×10^5^ CD4^+^CD45Rb^high^ T cells from BALB/c or from C57BL/6 were transferred i.p. into SCID (BALB/c background) or RAG mice (C57BL/6 background) as described [26]. SCID mice developed colitis within 10 to 12 weeks, whereas in RAG-1^−/−^ mice disease onset was accelerated and occurred already after 3 to 4 weeks.

### Cell Isolation and Purification

For analysis of homing receptor expression single cell suspensions were produced from MLN and spleen by teasing the cut organs through stainless steel meshes. After washing erythrocytes were lysed by hypotonic lyses.

For CD4 T cell isolation, cells were purified either by panning using anti-CD8 (53–672), anti-CD25 (PC/6), anti-Mac-1 (M1/70) and anti-FcR II/III (2.4G2) antibodies or by direct isolation of CD4^+^ cells by anti-CD4-FITC (GK1.5) and anti-FITC multisort-MACS beads to a purity of ≥ 98%. Naive CD4^+^CD62L^+^ cells were MACS-sorted with anti-CD62L microbeads to a purity of ≥ 98%. In some experiments CD25^+^ T cells were depleted before further enrichment of naive T cells.

MLN-derived DCs were prepared by positive selection of CD11c^+^ cells using anti-CD11c-MACS beads reaching a purity of about 95% of CD11c^+^ DCs.

For induction of SCID or RAG-1^−/−^ colitis CD4^+^CD45Rb^high^ T cells were enriched by CD4-FITC and anti-FITC-MACS beads. The CD45Rb^high^ population was sorted after staining with CD45Rb-PE by the Fluorescent activated cell sorter (FACS-DIVA; BD Biosciences, Heidelberg, Germany) to a purity of >95–99%.

### *In vitro* Stimulation of Naïve T Cells

For DC-dependent stimulation naive OVA-TCR^tg^ T cells from DO11.10 mice were activated by 0.5 µM OVA_323–339_ peptide (Biochemistry department, Charité, Berlin) in the presence of CD11c^+^ DCs at a T cell/DC cell ratio of 10∶1 and total cell number of 2×10^6^ cells/ml in complete RPMI 1640, containing 10% FCS and 10 µM 2-ME (Life Technologies). 1 µg/ml LPS (Sigma-Aldrich, Muenchen, Germany) from *E. coli* strain 055:B5 was added. Homing receptor expression was determined on day 5 after stimulation. Retinal (Sigma- Aldrich) was added at a concentration of 10 nM.

For polyclonal activation, sorted naive T cells from BALB/c mice were cultured on plates coated with anti-CD3 and anti-CD28 mAbs at 1×10^6^ cells/ml in the presence or absence of retinoic acid (10 nM). For Th1 polarizing conditions cultures were supplemented with recombinant murine IL-2 at 5 ng/ml, IL-12 at 5 ng/ml, IFNγ at 20 ng/ml (all recombinant cytokines: R&D Systems, Wiesbaden, Germany) and neutralizing anti-IL-4 mAb at 5 µg/ml. For Th0 conditions recombinant murine IL-2 at 5 ng/ml and neutralizing anti-IL-12 mAb, anti-IFNγ and anti-IL-4 mAb were added to the culture. Cells were removed from the stimulus after 72 h and rested for 2 days before analysis.

For homing studies, Th1 cells were generated from wild type and *Fut7^−/−^* mice by polyclonal activation by plate-bound anti-CD3/anti-CD28 stimulation in the presence of IL-12, IFNγ and anti-IL-4. After 72 h cells were removed from the stimulus and rested for 2 further days. On day 5 the cells were used for migration studies.

### Flow Cytometric Analysis

Cytometric analysis was performed using a FACSCalibur and the CellQuest software (BD Biosciences). P-selectin ligands were detected by P-selectin human IgG chimeric protein stained in HBSS containing Ca^2+^ and Mg^2+^and PE-conjugated anti-human IgG antibody. OVA-TCR^tg^ CD4^+^ T cells were identified using the clonotype-specific antibody KJ1.26.

For intracellular cytokine detection cells were stimulated with phorbol myristate acetate (PMA)/ionomycin (10 ng/ml; 500 ng/ml; Sigma-Aldrich) for 4 h with addition of Brefeldin A (10 µg/ml; Sigma-Aldrich) for the last 2 h. Afterwards, cells were surface stained for CD4 and selectin ligands, fixed in 2% paraformaldehyde (Sigma-Aldrich), permeabilized by 0.5% saponin (Sigma) and stained intracellularly for IFNγ. To prevent unspecific staining, anti FcRII/II (2.4G2) and whole rat IgG (Jackson Immuno Research) were added.

### Quantitative PCR

Total RNA was isolated with RNeasy Mini Kit and QiaShredder (Qiagen) and DNA removed with RNase-Free DNase Set (Qiagen). RNA was reverse transcribed by Superscript II Reverse Transcriptase (Invitrogen) using oligo(dT) and random hexamer primer (Qiagen). Quantitative PCR was performed on a Mx3000P or Mx3005P qPCR system (Agilent Technologies, Santa Clara, USA). Platinum SYBR Green qPCR Super-Mix-UDG (Invitrogen) was used together with the primers previously described for FucT-VII and C2GlcNAcT-I [27]. For detection of CCR9, PSGL-1 and FucT-IV mRNA the following primers were used: CCR9: forward: 5′-TGCCATGTTCATCTCCAACTG-3′, reverse: 5′-GAACTGGGTTCAGACAACTGTGG-3; PSGL-1: forward: 5′-GGGATGACGATTTTGAGGAC-3′, reverse: 5′-TCCTGTACCTGGGGCAGTAG-3′; FucT-IV: forward: 5′-GAGGTGGGTGTGGATGAACT-3′, reverse: 5′-TCGCTCCTGGAATAGAGGAA-3′.

### *In vivo* Homing Assay

The homing assay was performed as previously described [28]. In brief, Th1 cells were labeled with 20 µCi/ml ^51^Cr (Amersham Buchler) followed by incubation at 37°C for two hours in fresh medium and removal of dead cells with gradient centrifugation (17.1% isotonic Nycodenz, Nyegaard, Norway). 1–4×10^6^ labeled cells were injected into tail vein of mice. After three hours mice were sacrificed, several organs were removed and radioactivity of these organs and the remaining body was counted using a γ-counter (Wallac, Turku, Finland).

## Results

### Enhanced Frequency of P-lig^+^ CD4^+^ T Cells in MLN during Small and Large Intestinal Inflammation

**Figure 1:**
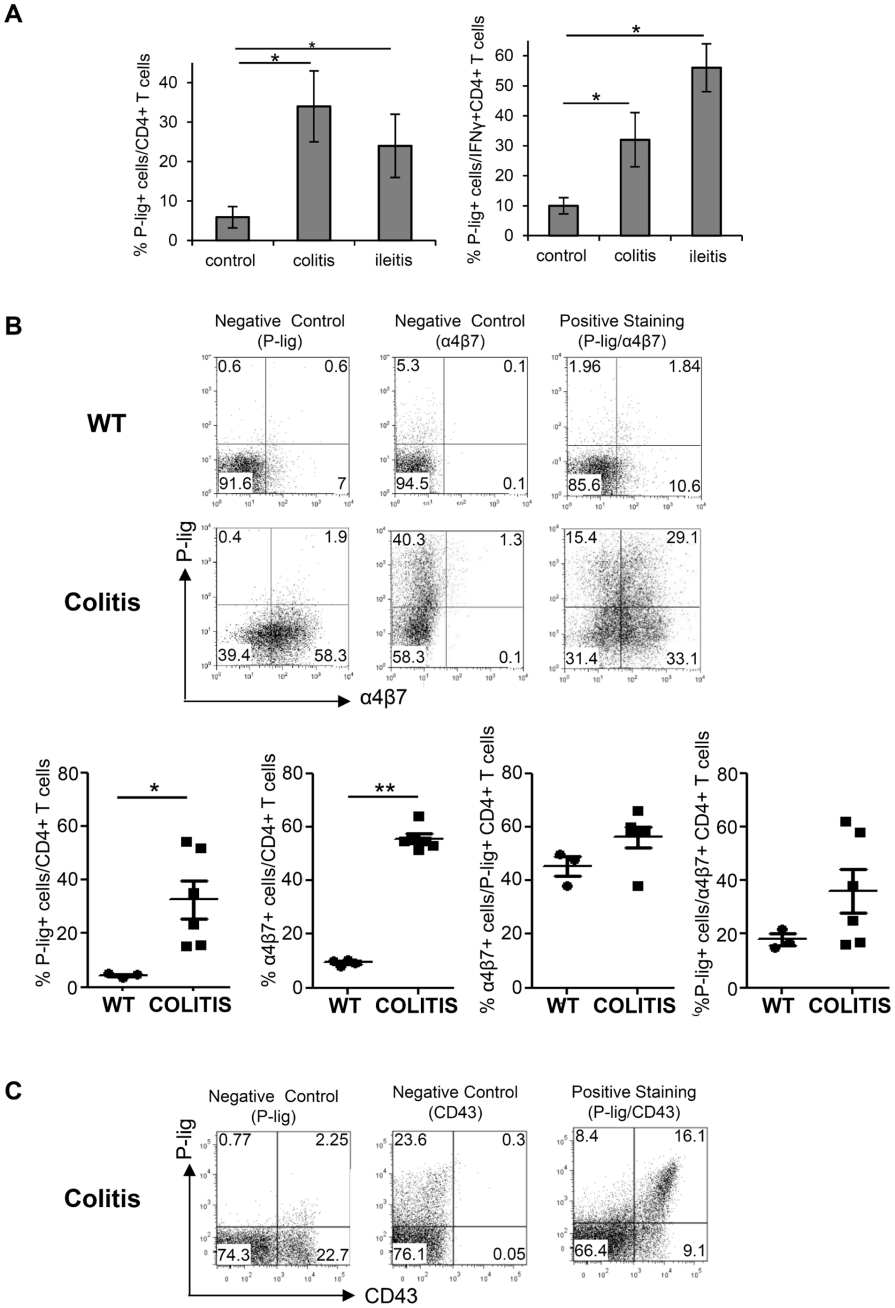
Coexpression of P-lig and α4β7 on a major subset of CD4^+^ T cells in MLN during intestinal inflammation. A) The percentage of P-lig^+^ cells/CD4^+^ T cells (left panel) and the percentage of P-lig^+^ cells/IFNγ^+^CD4^+^ T cells (right panel) in MLN of healthy mice (control), SCID mice after induction of colitis (colitis) and mice orally infected with 10 cysts of *T. gondii* (ileitis) is shown. Mean ± SD of four to six mice per group from two independent experiments is shown. B) In the upper panel, examples of control and positive stainings of P-lig^+^ and α4β7^+^ cells among CD4^+^ T cells from MLN of wildtype mice (WT) and RAG-1^−/−^ mice after induction of colitis (colitis) is shown. Lower panel shows the mean and individual measurements of the frequencies of P-lig^+^ cells/CD4^+^ T cells and α4β7^+^ cells/CD4^+^ T cells acquired in two independent experiments. Among P-lig^+^ cells the frequency of α4β7^+^ co-expressing cells was determined as well as the frequency of P-lig^+^ cells among α4β7^+^ T cells. C) Control and positive stainings of the 130 kDa isoform of CD43 and P-lig gated on CD4^+^ T cells from pooled cells of MLN of four mice with colitis induced as in B (one representative example of two independent experiments). *p<0.05; **p<0.01; Mann Withney U test.

Induction of P-lig has been described during inflammation of the large intestine [22] and also within the small intestine after intraperitoneal immunization [23]. To analyze whether P-lig is commonly induced during intestinal inflammation we determined the P-lig expression of Th1 effector cells in MLN during inflammation of either the large or the small intestine. Large intestinal inflammation was induced by transfer of naive T cells into SCID mice [29] while small intestinal inflammation, i.e. a pan-ileitis, was induced by oral infection of wildtype (WT) mice with *T. gondii* [30], [31]. During colitis but also during ileitis, more than 25% of the CD4^+^ T cells in MLN expressed P-lig which was significantly higher than under homeostatic conditions (Figure 1A). Also if we determined specifically the percentage of P-lig^+^ cells among Th1 effector cells identified by IFNγ-expression a drastic increase in the frequency of P-lig^+^ cells was observed during both, colitis and ileitis, compared to homeostatic conditions (Figure 1A). This suggests that during intestinal inflammation P-lig is strongly induced on newly generated Th1 effector cells.

### P-lig and α4β7 are Co-expressed on MLN CD4^+^ T Cells

RA, the main inducer of α4β7, was shown to inhibit the expression of E- and P-lig *in vitro* [6]. To analyze whether P-lig induction under intestinal inflammation occurs in parallel to α4β7 induction and hence in the presence of RA, we analyzed P-lig and α4β7 expression simultaneously in MLN of RAG-1^−/−^ mice after induction of colitis by transfer of CD45Rb^high^ T cells in comparison to healthy C57BL/6 mice. In addition to the enhanced frequencies of P-lig^+^ CD4^+^ T cells we observed a dramatic increase in the frequency of α4β7 CD4^+^ T cells in the colitis mice indicating indirectly that the majority of effector cells had been exposed to RA during priming (Figure 1B). Also among P-lig^+^ CD4^+^ T cells, the majority, i.e. about 60%, co-expressed α4β7 suggesting that these cells have been exposed to RA during priming as well. Among the few P-lig^+^ CD4^+^ T cells found in healthy C57/Bl6 mice more than 40% co-expressed α4β7 showing that co-induction of α4β7 and P-lig can also occur under non-lymphopenic conditions (Figure 1B). Among the enlarged fraction of α4β7^+^ CD4^+^ T cells found during colitis a significant percentage, i.e. about 35% co-expressed P-lig (Figure 1B).

This suggests that P-lig and α4β7 are co-induced under inflammatory conditions within the intestine. To determine whether the P-selectin-binding activity of CD4^+^ effector cells generated during intestinal inflammation depends on C2GlcNAcT-I activity we analyzed the expression of the 130 kDa isoform of CD43 – a C2GlcNAcT-I-dependent epitope which is specifically recognized by the mAb 1B11 and used as a surrogate marker of C2GlcNAcT-I activity [32], [33]. As shown in Figure 1C, essentially all P-lig^+^ CD4^+^ cells within MLN of colitis mice co-expressed the 130 kDa isoform of CD43 indicating C2GlcNAcT-I activity in these cells.

### P-lig Expression on T Cells Supports Recruitment of CD4^+^ Effector T Cells into the Inflamed Large Intestine but not into the Inflamed Small Intestine

**Figure 2:**
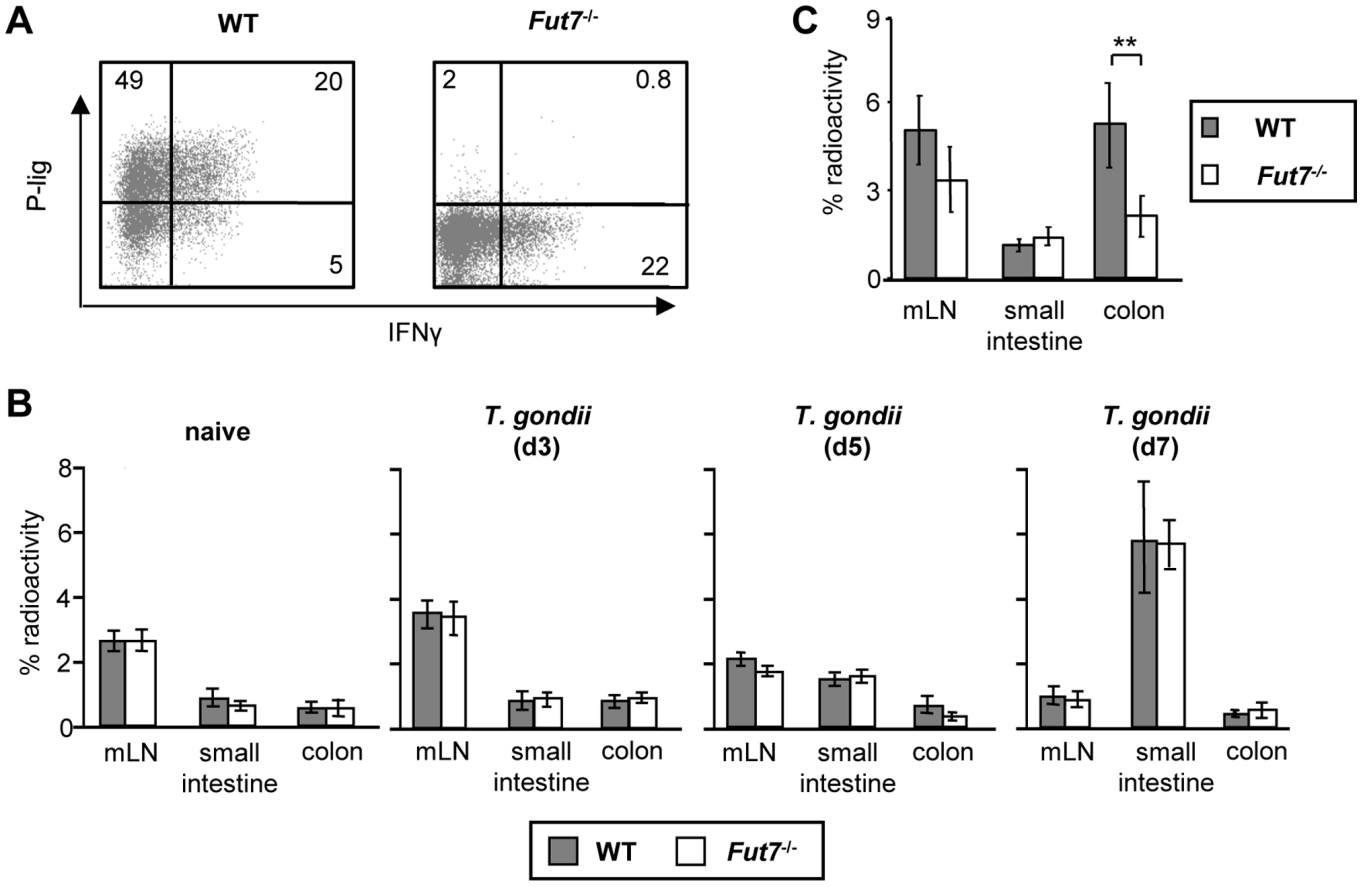
P-lig expression can support recruitment of Th1 cells into the inflamed colon but not into the inflamed small intestine. A) Naive T cells from wiltype (WT) and *Fut7^−/−^* mice were differentiated in vitro under Th1 polarizing conditions in the absence of RA. P-lig and PMA/ionomycin-induced IFNγ expression of Th1 cells generated from *Fut7^−/−^* and WT mice is shown. B) ^51^Cr-labeled Th1 cells generated from WT or *Fut7^−/−^* mice were transferred into naive mice or mice infected orally with 100 cysts of *T. gondii* three, five or seven days before. Radioactivity (mean ± SD ) per organ was determined three hours after transfer of radioactive T cells and is given as the percentage of total recovered radioactivity (n = 5 animals/group). The experiment was repeated on d7 with 5 animals/group with similar results. C) ^51^Cr-labeled Th1 cells from WT or *Fut7^−/−^* mice were injected into SCID mice with established colitis induced by transfer of CD4^+^CD45Rb^high^ cells. The percentage (mean and SD) of recovered radioactivity per organ of one representative experiment out of 3 independent experiments with 4–6 mice/ group is shown. **p<0.01; Mann Whitney U test.

Now, we aimed to analyze whether selectin ligand expression is at all required for Th1 cells to enter inflamed intestinal sites. Therefore, we generated Th1 cells from wildtype (WT) or *Fut7^−/−^*deficient mice. In *Fut7^−/−^* Th1 cells P-lig [15]) expression is abolished while effector functions, such as IFNγ production, are unaffected (Figure 2A). To study the impact of P-lig for T cell recruitment into small intestinal inflammation WT or *Fut7* ^−/−^ Th1 cells were radioactively labeled and transferred into untreated recipient mice or mice orally infected with *T. gondii* either three, five or seven days before. *Toxoplasma gondii* infected mice were randomly assigned at the day of the homing experiment to the groups either receiving Th1 cells from WT or *Fut7^−/−^* mice. Infection of mice with *T. gondii* elicited a strong immigration of Th1 cells into the small intestine on day 7 after infection, the day when inflammatory responses and development of small intestinal pathology peak [30], [31] as indicated by increased recovered radioactivity compared to non-infected gut (Figure 2B). However, *Fut7^−/−^* Th1 cells lacking P-lig expression entered the small intestine equally well as WT Th1 cells suggesting that P-lig expression on T cells is dispensable for the recruitment of Th1 cells to the inflamed small intestine. To analyze whether P-lig can support recruitment into the inflamed colon WT and *Fut7^−/−^* Th1 cells were transferred into SCID mice in which colitis was induced before by transfer of CD45Rb^high^ T cells from WT mice. Migration of *Fut7^−/^* ^−^ Th1 cells into the inflamed colon was reduced by about 50% compared to WT Th1 cells (Figure 2C) suggesting that the interaction of P-lig with its ligand P-selectin can indeed support immigration of Th1 cells into the inflamed colon.

### Treatment with LPS Enhances the Induction of P-lig on CD4^+^ T Cells by MLN-derived DCs

**Figure 3:**
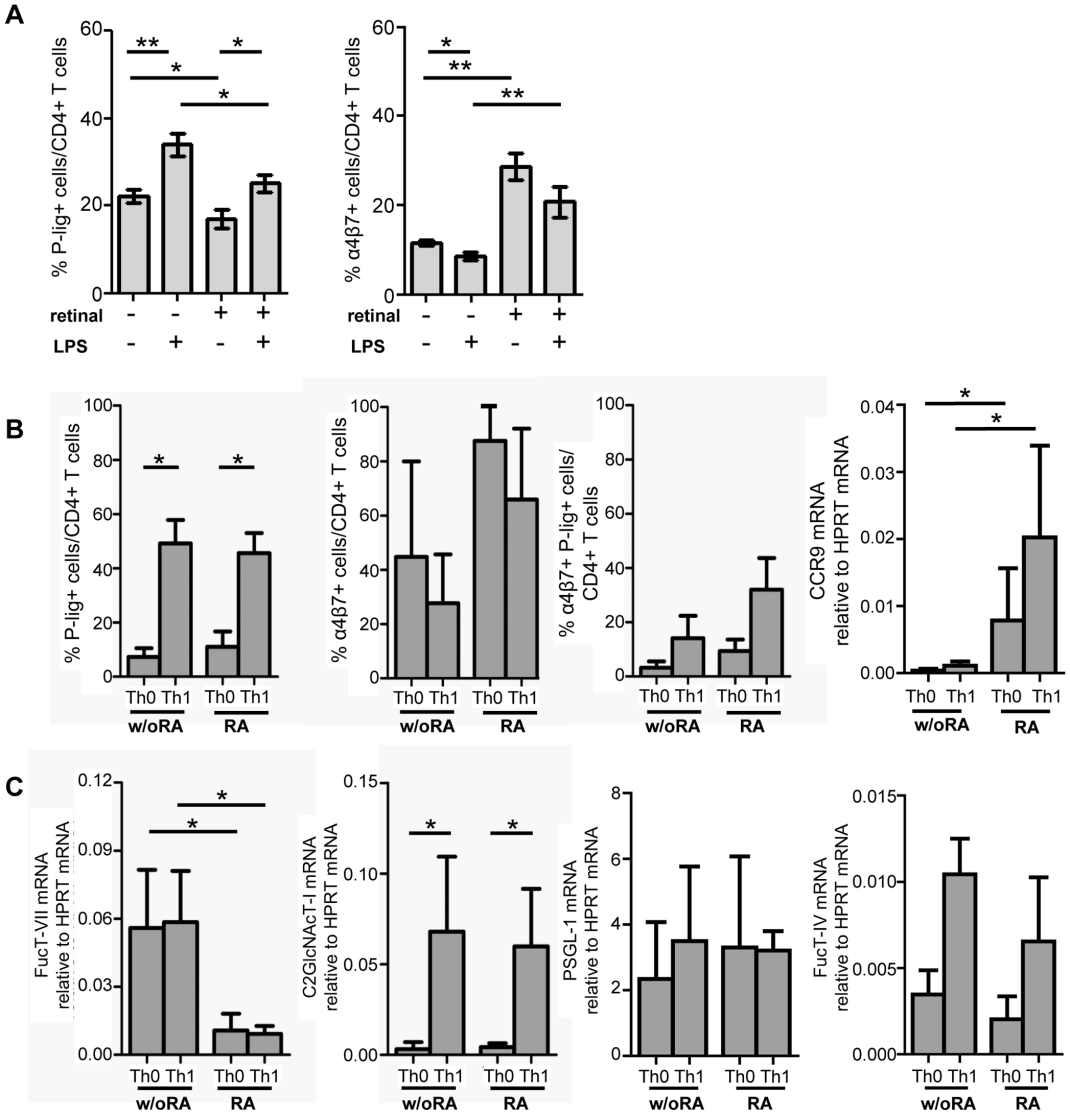
Inflammatory stimuli increase P-lig but reduce α4β7induction on CD4^+^ T cells even in the presence of RA. A) Naive OVA-TCR^tg^ CD4^+^ T cells were activated with OVA_323–339_ peptide and CD11c^+^ DCs from MLN in the presence or absence of LPS and presence or absence of 10 nM retinal. Percentage of P-lig^+^/CD4^+^ T cells and α4β7^+^/CD4^+^ T cells (mean + SD of n = 6 from two independent experiments) was determined on d5 after activation. B) Naive CD4^+^ T cells were activated by plate bound antiCD3/antiCD28 in the presence or absence of polarizing cytokines and 10 nM retinoic acid. The frequency of P-lig^+^, α4β7^+^ as well as double-positive CD4^+^ T cells was determined on day 4 by FACS while CCR9 expression was determined on mRNA level (mean + SD, n = 4 from four independent experiments). C) FucT-VII and C2GlcNAcT-I mRNA as well as PSGL-1 and FucT-IV mRNA expression were determined on day 4 after activation (mean + SD of four independent experiments). *p<0.05; **p<0.01; Mann Whitney U test (for A–C).

To clarify the mechanism of P-lig induction within the gut environment, i.e. in the presence of RA, we first analyzed whether bacterial stimuli affect the capacity of MLN-DCs to induce P-lig on T cells. Therefore, we isolated CD11c^+^ DCs from MLN and used those for activation of OVA-transgenic T cells by OVA in the presence or absence of LPS. LPS significantly enhanced the induction of P-lig on CD4^+^ T cells cultured in the presence of MLN-derived DCs but slightly reduced induction of α4β7 (Figure 3A). Addition of retinal which is metabolized by MLN-DCs to retinoic acid was accompanied by a strongly increased induction of α4β77 on CD4^+^ T cells whereas P-lig induction was only slightly reduced. The additional inducing effect of LPS on P-lig expression was retained in cultures containing retinal.

LPS treatment of MLN-derived DCs could affect the capability of the DCs to convert retinal or it could induce production of inflammatory cytokine, in particular IL-12, which is known to induce P-lig. To determine if Th1 polarizing cytokines can induce P-lig expression even in the presence of RA, we activated naïve T cells by anti-CD3/CD28 in the presence or absence of RA and in the absence or presence of Th1-polarizing cytokines, i.e. IL-12, IFNγ and anti-IL-4 (Figure 3B). In the absence of polarizing cytokines, i.e. under Th0 conditions, no matter whether RA was present or not, low induction of P-lig was seen. α4β7, expressed at intermediate levels in the absence of RA, increased upon exposure to 10 nM RA. Similarly, presence of RA increased CCR9 mRNA expression under non-polarizing Th0 and Th1-polarizing conditions. In the presence of Th1 cytokines, high levels of P-lig were induced even in the presence of RA. α4β7 induction was slightly reduced under these conditions. The frequency of P-lig/ α4β7 double positive CD4^+^ T cells increased to about 40% in the presence of RA and Th1-polarizing conditions. Analysis of FucT-VII and C2GlcNAcT-I mRNA levels showed that although RA almost completely abrogated FucT-VII induction, C2GlcNAcT-I mRNA induction was not significantly reduced by RA and reached similar levels as under Th1 conditions in the absence of RA (Figure 3C). PSGL-1 levels were unaffected by RA treatment. Also expression levels of FucT-IV induced under Th1 conditions were unchanged upon RA treatment. This suggests that unimpaired C2GlcNAcT-I expression determines P-lig expression under these conditions even in the presence of low FucT-VII induction.

## Discussion

In this study we confirm that P-ligs are induced on a significant proportion of CD4^+^ T cells during inflammation within the intestine. During small and large intestinal inflammation we observed a high frequency of P-lig expressing T cells in the draining MLN excluding a major impact of the specific intestinal location on this induction. As during intestinal inflammation the majority of P-lig^+^ T cells found within the MLNs co-expressed α4β7, intestinal priming of these cells is very likely. This suggests that induction of P-ligs is effective under inflammatory conditions even in the intestinal environment which primarily supports induction of gut-specific homing molecules [1].

The functional role of P-selectin-P-lig interactions for mucosal inflammation is controversially discussed [34]. Bonder et al. showed E- and P-selectin-dependent interactions of Th1 and Th2 cells within small intestine in response to intraperitoneal TNF and IL-4 treatment by intravital microscopy [35]. In models of chronic ileitis like the SAMP/Yit model, administration of a mAb directed against P-selectin-glycoprotein-1 (PSGL-1, 2PH1), which primarily affects P-selectin-dependent adhesion, attenuated disease [36]. Interestingly, PSGL-1 is displayed in this model by the small intestinal vasculature and only vascular expression was critical for disease development whereas it was dispensable on hematopoetic cells [37].

In accordance with this latter study, deficiency in selectin-ligand expression on Th1 cells did not abrogate recruitment of the Th1 cells into the inflamed small intestine during oral infection with *T. gondii* as tested here. In addition to the proposed interaction of leukocytes with vascular PSGL-1, immigration of the *in vitro* generated Th1 cells could also be guided by interaction of α4β7 with MAdCAM as these cells also express α4β7 (Figure 3B). In conclusion, both the SAMP/Yit model, a mixed Th1/Th2 model [38] and the Th1 model of *Toxoplasma gondii*-induced ileitis [24], do not require T cell-expressed selectin ligands for entry of Th1 cells into the inflamed small intestine. This might suggest that selectin ligands on T cells are in general dispensable for their entry into the small intestine in T cell-dependent ileitis.

In contrast to the small intestine, we found about 50% impairment of the recruitment of *Fut7* ^−/−^ Th1 cells into the inflamed colon. Thus, P-selectin-dependent homing mechanisms contribute to recruitment into the inflamed colon.

In a DTH model we found that immigration of *Fut7* ^−/−^ Th1 is reduced by more than 90% compared to WT Th1 cells [39]. This suggests that within the inflamed skin, entry of Th1 cells almost completely depends on E- and P-selectin-dependent adhesion whereas in colitis, P-lig-dependent migration accounts only partially for inflammatory T cell recruitment. In line with our study, blockade of P-selectin dependent interaction with the PSGL-1 mAb can reduce leukocyte recruitment in the acute dextran sodium sulfate (DSS) colitis model which also attenuates disease [40]. In contrast, PSGL-1 deficient CD4^+^ T cells were able to induce colitis in the transfer colitis model as used here [41]. This suggests that P-selectin dependent adhesion mechanisms rather complement or synergize during inflammation with the α4β7-dependent adhesion system of the intestine. Thus, transfer of naive T cells from *Itgb7^−/−^* mice results in strongly delayed, although not complete prevention of colitis confirming the importance of the α4β7-dependent adhesion for rapid and efficient localization of T cells in intestinal tissues [42].

By analyzing the frequency of P-lig^+^ T cells among cytokine producing cells within the spleen and lung during influenza virus and *Nippostrongylus* infection and within the liver during oral *T. gondii* infection we have previously found, that P-lig expression is particularly high among cytokine producing cells at the site of inflammation [10]. Similar to that, we found a high proportion, i.e. about 50%, of P-lig^+^ T cells among IFNγ-producing T cells within the MLN during *T. gondii* infection. This suggests that the local inflammatory mediators present during ileitis and colitis not only promote Th1 differentiation but also induction of P-lig expression, even in the intestine. In this line, Haddad et al. showed that P-lig expression on T cells primed within MLN after antigen-specific i.p.-immunization in the presence of CFA, a strong trigger of Th1 immunity, is abrogated by IL-12 blockade [23]. Innate, in particular dendritic cells are activated by bacterial structures and there is a debate whether Toll like receptor (TLR) triggering can modulate the retinal-converting capacity of DCs. In fact, a decrease in the aldehyde dehydrogenase (ALDH) activity has been described in MLN DCs from *Myd88^−/−^ Trif^−/−^* mice and from germfree mice suggesting that bacterial stimuli promote expression of ALDH [43]. However, among several TLR ligands only TLR 1/2 ligand Pam_3_CSK_4_ but not LPS strongly upregulated ALDH activity of mucosal DCs from mice housed under specific pathogen-free conditions [44]. Our results suggest that LPS might even suppress ALDH activity since expression α4β7 was slightly reduced upon LPS exposure of MLN-DCs. However, as we observed a strong increase in the frequency of α4β7 CD4^+^ cells during colitis this effect seems to be negligible in vivo. The high percentage of P-lig^+^ T cells in MLN co-expressing α4β7 rather suggests that inflammatory stimuli promoted P-lig induction despite the presence of RA. In line with this, we observed strong induction of P-lig under Th1-polarizing conditions in vitro even in the presence of retinoic acid. Thus, this suggests that enhanced P-lig expression upon intestinal inflammation is primarily stimulated by enhanced local cytokine production rather than by modulation of the retinal-converting capacity.

The generation of P-lig epitopes in CD4^+^ T cells depends on induction of FucT-VII and C2GlcNAcT-I [14], [15]. In accordance with published data, we found strong inhibition of FucT-VII mRNA expression by RA [4], [6]. However, IL-12-induced C2GlcNAcT-I expression was almost unaffected by retinoic acid and obviously promoted P-lig expression even in the presence of low FucT-VII expression. Involvement of C2GlcNAcT-I is also suggested by our in vivo data, as we found high expression of the core-2-dependent 130 kDa isoform of CD43. However, as *Fut7^−/−^* Th1 cells lack P-lig expression (Figure 3) residual, low level expression of FucT-VII is required for P-lig generation.

Altogether our data suggest that inflammatory stimuli can overrule tissue-specific homing pathways in the gut. P-lig expression is achieved under these conditions by unimpaired induction of C2GlcNAcT-I by IL-12 even though FucT-VII induction is suppressed in a retinoic acid-dependent way.
